# Spatial organisation of fungi in soil biocrusts of the Kalahari is related to bacterial community structure and may indicate ecological functions of fungi in drylands

**DOI:** 10.3389/fmicb.2024.1173637

**Published:** 2024-04-25

**Authors:** David R. Elliott, Andrew D. Thomas, Stephen R. Hoon, Robin Sen

**Affiliations:** ^1^Environmental Sustainability Research Centre, University of Derby, Derby, United Kingdom; ^2^Department of Geography and Earth Sciences, Aberystwyth University, Aberystwyth, United Kingdom; ^3^Department of Natural Sciences, Manchester Metropolitan University, Manchester, United Kingdom

**Keywords:** biocrust, fungi, bacteria, Kalahari, soil, dryland, carbon, biogeochemistry

## Abstract

Biological soil crusts, or biocrusts, are microbial communities found in soil surfaces in drylands and in other locations where vascular plant cover is incomplete. They are functionally significant for numerous ecosystem services, most notably in the C fixation and storage due to the ubiquity of photosynthetic microbes. Whereas carbon fixation and storage have been well studied in biocrusts, the composition, function and characteristics of other organisms in the biocrust such as heterotrophic bacteria and especially fungi are considerably less studied and this limits our ability to gain a holistic understanding of biocrust ecology and function. In this research we characterised the fungal community in biocrusts developed on Kalahari Sand soils from a site in southwest Botswana, and combined these data with previously published bacterial community data from the same site. By identifying organisational patterns in the community structure of fungi and bacteria, we found fungi that were either significantly associated with biocrust or the soil beneath biocrusts, leading to the conclusion that they likely perform functions related to the spatial organisation observed. Furthermore, we showed that within biocrusts bacterial and fungal community structures are correlated with each other i.e., a change in the bacterial community is reflected by a corresponding change in the fungal community. Importantly, this correlation but that this correlation does not occur in nearby soils. We propose that different fungi engage in short-range and long-range interactions with dryland soil surface bacteria. We have identified fungi which are candidates for further studies into their potential roles in biocrust ecology at short ranges (e.g., processing of complex compounds for waste management and resource provisioning) and longer ranges (e.g., translocation of resources such as water and the fungal loop model). This research shows that fungi are likely to have a greater contribution to biocrust function and dryland ecology than has generally been recognised.

## 1 Introduction

Biological soil crusts (biocrusts) cover approximately 50% of the soil surface in drylands (Garcia-Pichel et al., 2003) and 30% of the global land area (Beraldi-Campesi, 2013). Biocrusts are composed of microbes in a fine spatial arrangement at the soil surface, typically occupying less than the upper 1 cm (Elliott et al., 2014). In most drylands (arid, semi-arid and dry sub-humid regions with seasonal/annual moisture deficits), this upper centimetre of soil contains the highest concentration of organic C in the soil profile (Thomas, 2012). It is exposed to direct sunlight, and experiences diurnal temperature extremes with only intermittent periods of hydration (Pointing and Belnap, 2012). Dryland soil surfaces supporting biocrusts are therefore a unique and important interface between the atmosphere and soil. Although we know an increasing amount about their microbial inhabitants (see Weber and Büdel, 2016 and the references therein for a comprehensive summary), we know less about microbial adaptations to the extremes of their habitat (Green and Proctor, 2016), and even less about interactions between their constituent microbial groups and neighbouring plants or their contributions to ecosystem function (Zhang et al., 2016). Dryland soil microbiology studies are also rare in the global literature and are under-represented in DNA sequence databases especially in Africa (e.g., MG-RAST and NCBI SRA). Our overall understanding of dryland ecosystems is consequently limited by a lack of knowledge concerning microbial ecology of the soil surface.

It is clear that microbes in biocrusts perform multiple important ecological functions such as fixation of atmospheric carbon and nitrogen and production of polymeric substances (e.g., extracellular polysaccharide and glycoproteins) that enhance soil aggregate stability by binding soil mineral grains together, significantly reducing soil erodibility (Bowker et al., 2008; Mager and Thomas, 2011). These poikilohydric microbial communities even occur in hyper-arid areas devoid of vascular plants where many of their ecosystem functions are analogous to those of plants (Ferrenberg et al., 2017). However, microbial biocrusts also dominate the interspaces between plants and trees in semi-arid ecosystems (Lan et al., 2021) where they appear to influence successful plant establishment (Ochoa-Hueso et al., 2018).

Microbe-mediated biocrust functions may substantially influence global scale biogeochemical cycles and climate (e.g., Sancho et al., 2016) but we presently lack sufficient knowledge of microbial communities to properly assess the ecological roles of biocrusts in drylands (Ferrenberg et al., 2017). Phototrophs, specifically cyanobacteria and eukaryotic algae, have been the focus of biocrust research, and their role as primary producers in crusted soils is well established (e.g., Büdel et al., 2016). To gain a comprehensive understanding of the ecology and function of biocrusts, however, other organisms that form part of the biocrust community must also be considered (Beraldi-Campesi, 2013; da Rocha et al., 2015). For example, the carbon cycle is not exclusively controlled by the photosynthetic community members, but also the relative activities of heterotrophic organisms (Collins et al., 2008) and the dynamics of competition, predation and disease within biocrust communities. Recent research is just beginning to provide insights into the potential functional implications of heterotrophic biocrust communities (Bethany et al., 2022).

Collins et al. (2008) proposed that fungi play a critical role in carbon and nitrogen cycling in dryland ecosystems, performing translocation functions between biocrusts and plants, which they describe as a “fungal loop.” Rudgers et al. (2018), however, assert that conclusive evidence for the fungal loop has, as yet, not been found for any dryland ecosystem. They propose criteria with which to test the hypothesis, which include the direct observation of fungal networks connecting plants with biocrusts. As well as interacting with plants, biocrust communities must also be able to independently meet their own resource requirements, either on a permanent basis in places lacking plants, or on a temporary basis according to environmental constraints upon plant activity. A complete understanding of the hypothesised fungal loop model must therefore also include an examination of whether biocrusts can establish a microbial loop within the soil that is independent of plants.

Elucidating a process-based understanding of microbial interactions in biocrusts is challenging due to the small scale of biocrusts, their heterogeneity, and the typically complex non-linear responses of biocrust communities to changing environmental conditions (e.g., Collins et al., 2008; Pointing and Belnap, 2012; Thomas et al., 2022). However, the outcome of community interactions is encoded in the spatial organisation of community structure which can be extensively characterised using culture-independent DNA sequencing approaches. Spatial patterns of community members can be logically predicted based upon expected processes which are required to sustain the life of biocrust, thus providing a basis to form and test hypotheses about microbial community function in biocrusts. For example, the fungal loop model predicts that fungi will occur not only within biocrusts but extend beneath to meet with plant roots (Collins et al., 2008). Accordingly in this research, we tested for expected community structure arrangements that are consistent with the fungal loop model, and we also tested for correlation between bacterial and fungal community structure which would be consistent with symbiotic fungal participation in the community function of biocrusts.

In previously published research from southwest Botswana, we showed that biocrust bacterial communities (0–1 cm depth) differ from bacterial communities in the underlying soil (1–2 cm depth; Elliott et al., 2014). In addition, biocrust inhabiting bacterial communities varied in relation to predominant vegetation types, which are determined in part by their distinctive microclimates (Thomas et al., 2018) and by grazing pressure driving shifts from palatable to less palatable grasses (Lan et al., 2021). In this research we sought to identify fungi which may engage in short-range and long-range interactions with biocrusts, by searching for taxon distribution patterns which are consistent with hypothetical functional roles (Table 1).

**Table 1:** Hypothetical ecological functions of fungi in biocrusts, and the expected distribution of fungi fulfilling these roles.

Measured community property	Short-range interactions with biocrust	Long-range interactions with biocrust
	e.g., cross-feeding, waste processing, physical protection	e.g., translocation of resources through soil
Fungal taxa distribution by vegetation cover type (see Figure 4)	Taxa more abundant in open areas with photosynthetic biocrusts	Varies dependent on function (not tested)
Fungal taxa distribution by depth in open areas with biocrust (see Figure 5A)	Taxa are more abundant in the biocrust compared to beneath it.	Taxa are similarly abundant within and beneath the biocrust

We have two main hypotheses in support of the idea that fungi interact with the bacterial community within the biocrust and extend beyond the biocrust to enhance resource provisioning.

First, we hypothesise that the bacterial and fungal community composition in biocrusts will be correlated with each other because they function together as an ecological system for enabling short-range interactions that involve physical contact or interactions with the excreted products of organisms in the soil. These hypothetical short-range interactions could include physical protection and cross-feeding where the waste or excretion of one organism is the food for the other. The correlation of community structure can be evaluated by representing the bacterial and fungal communities as separate distance matrices, then checking for correlation between them, which would indicate that they are linked and not independent of each other (see section 2.4 for the full details). In contrast, we expect that the bacterial and fungal communities beneath biocrusts, and in areas lacking biocrusts, will not be correlated with each other in the same way.

Second, we hypothesise that some fungi will be associated with biocrusts but not limited in extent to the surface layer of the biocrust. The presence of fungi associated with biocrusts which extend beneath the biocrust would be consistent with the fungal loop hypothesis, providing evidence supporting a potential role of fungi in connecting the biocrust to the surrounding soil and plant roots for the purpose of translocating resources such as water and minerals.

## 2 Materials and methods

### 2.1 Study site

**Figure 1:**
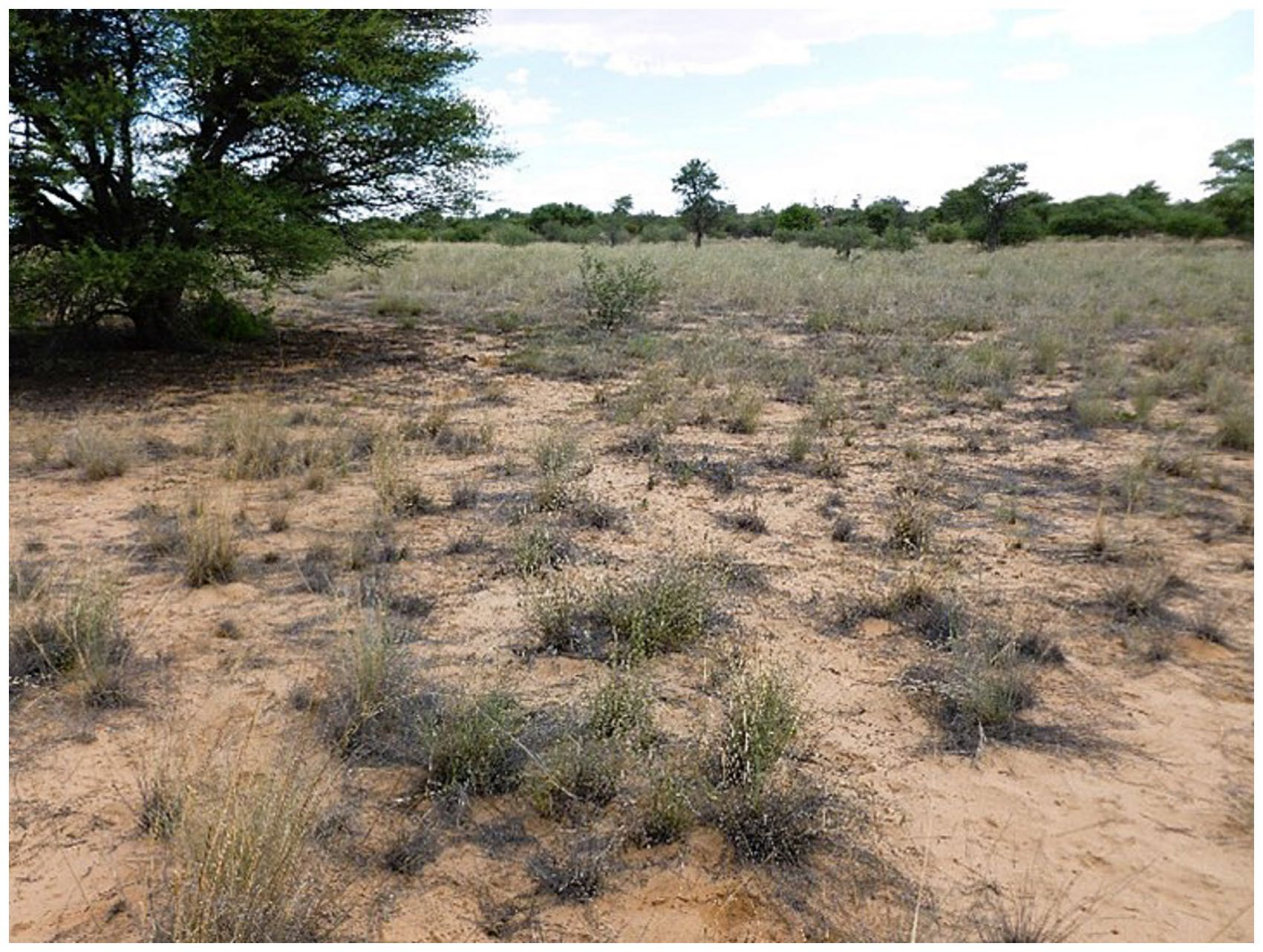
Mixed tree, shrub and grass rangeland at the study site. Extensive unvegetated soil patches between grasses are common and support the development of biocrusts. Photographs of individual vegetation types can be found in Elliott et al. (2014).

The study site is a semi-arid Kalahari rangeland in southwest Botswana (25°56′51″S, 22°25′40″E), consisting of open fine-leafed savanna with a mixture of perennial (*Eragrostis*) and annual (*Schmidtia*) grasses, woody shrubs [*Acacia mellifera* (Vahl) Benth and *Grewia flava* DC] and trees, predominantly *Acacia erioloba* E. Mayer (Figure 1). The *c*. 3 ha site is fenced, and livestock grazing was excluded for the duration of the study. Mean annual precipitation is 334 mm and air temperatures range from maxima frequently in excess of 40°C to below freezing. Soils are fine sand-sized, weakly acidic (pH 5.8 ± 0.2) Arenosols, locally known as Kalahari Sands. Soil carbon and nitrogen content is low, typically less than 1% and 0.1% w/w, respectively (Elliott et al., 2014; Supplementary Data Sheet 1). In lightly grazed areas, around 80% of the surface is covered in a 3–4 mm deep soil biocrust but cover declines rapidly with the frequency and intensity of grazing. Three broadly different biocrust types have been recorded in the area based on macroscopic morphology (Thomas and Dougill, 2006), carbon and nitrogen content (Lan et al., 2021) and bacterial communities (Elliott et al., 2014; Lan et al., 2021). These are a weakly consolidated crust with no surface discolouration (type 1); a consolidated crust with a black or brown speckled surface (type 2); and a crust with a bumpy surface and intensely coloured black/brown surface (type 3). Type 1 and type 2 biocrusts were present in the grass interspaces. Soils under shrubs were crusted but with very low levels of cyanobacteria, and soils under trees were completely unconsolidated, and lacked the abundance of cyanobacteria which is typically observed in biocrusts (Elliott et al., 2014; Lan et al., 2021). Crusted soils under shrubs also contain greater concentrations of carbon and nitrogen (Lan et al., 2021). In order to characterise fungal communities and cross-domain bacterial-fungal relationships, the same biocrust and soil samples from the bacterial community study (Elliott et al., 2014) were analysed in this study.

### 2.2 Sampling and DNA sequencing

Samples were collected from grass interspaces and under the canopy of trees and shrubs, with the objective being to sample the inter-space soil rather than the plant-associated soil. Samples from depth 0–1 cm contained the biocrust except in tree zones where biocrust was not present (Elliott et al., 2014). Sampling was performed aseptically in November 2011 at the end of the dry season and in March 2012 at the end of the wet season, and all soils were dry at the time of sampling (<0.5% w/w water content). Equal sampling in two seasons was carried out to ensure results were robust to possible seasonal effects, although it was not our intention to investigate seasonal effects.

Sampled locations in the first season campaign were selected from available sites that met selection criteria which included, no other sampling site of same type within 20 m, no other sampling site within 5 m, absence of other vegetation type within 2 m, no vegetation within 1 m, no unusual features such as animal borrows. In the second season campaign, sampling was carried out in adjacent locations, at least 2 m from previous sampling locations. Samples are identified throughout in terms of the nearby vegetation (4 types), soil depth (2 depths), and season (2 seasons; Elliott et al., 2014). The full factorial sampling design had a replication level of 3, yielding 48 samples in total.

Samples were collected by digging a pit, cutting clean faces with sterile tools, then removing samples using sterile spatulas. The total size of each sample was about 10 g and this was thoroughly homogenised before DNA extraction from a 400 mg sub-sample. This study used the same DNA extractions described in Elliott et al. (2014). In brief, DNA extraction was undertaken within 18 h of collection using a Mobio Powersoil DNA extraction kit. Sequencing of the fungal ribosomal ITS1 was performed by Research and Testing Laboratory (Lubbock, TX, United States) using a Roche 454 FLX instrument with Titanium reagents (Dowd et al., 2008). The ITS primers ITS1F (CTTGGTCATTTAGAGGAAGTAA; Gardes and Bruns, 1993) and ITS1R (TCCTCCGCTTATTGATATGC; White et al., 1990) were used in conjunction with an 8 bp barcode to enable multiplexing of samples on the sequencer.

### 2.3 Bioinformatics

Sequence data were processed using USEARCH version 10.0.240 (Edgar, 2013) for quality control, chimera removal, operational taxonomic unit (OTU) clustering, and OTU table generation. Quality control parameters for USEARCH included a truncation length of 340 bases, and a maximum expected error rate of 1.0. ITSx (Bengtsson-Palme et al., 2013) was used to remove non-fungal sequences and to trim sequences so that they contained only the target fungal ITS1 region. A 97% sequence similarity was used to define OTUs and the UNITE database (Kõljalg et al., 2013) was used for taxonomic classification (UNITE version 9; 29 November 2022). Taxonomy was assigned using the BLAST method (Altschul et al., 1990) implemented in QIIME2 (Bolyen et al., 2019). Data manipulations and statistical analyses were performed using R (R Core Team, 2023) and the phyloseq package (McMurdie and Holmes, 2013) for R.

### 2.4 Statistical analyses

To determine whether fungal communities differ with respect to nearby vegetation, depth, or sampling month a permutational multivariate analysis of variance test was carried out using the adonis2 function of R package Vegan (Oksanen et al., 2017). A constrained correspondence analysis (CCA) was used to visualise community features which specifically relate to nearby vegetation type or depth, based on the Bray-Curtis community dissimilarity, and performed using the phyloseq (McMurdie and Holmes, 2013) wrapper to the Vegan package (Oksanen et al., 2017) for R.

To investigate whether fungal communities in biocrusts are structured in-concert with the respective bacterial communities, we compared ecological composition matrices using Mantel tests. These used Bray-Curtis community dissimilarity matrices for bacteria (Elliott et al., 2014) and fungi (this study) which were compared with 999 permutations after checking for homogeneity of group dispersions, as implemented in the Vegan package (Oksanen et al., 2017). These tests for community correlation were performed on different sub-sets of the data to determine whether community correlation between bacteria and fungi is related to the presence of photosynthetic biocrusts.

We used the model based DESeq2 approach (Love et al., 2014) to identify candidate OTUs with spatial distributions that are consistent with functional roles within the biocrust or extending beyond the biocrust (Table 1). To identify OTUs possibly interacting at short-range within the biocrust we targeted fungi present in all grass interspace surface samples (these areas have cyanobacterial biocrust) but absent or very rare in all subsoil samples. To identify OTUs which may be interacting with the biocrust at long-range we targeted fungi which are present in both the soil surface (0–1 cm) and subsoil (1–2 cm) and are disproportionately abundant at sites with cyanobacterial biocrust compared to sites without cyanobacterial biocrust. The DESeq2 technique includes correction for multiple testing and is highly efficient on data preservation because it eliminates the need to perform rarefying or normalisation of count data (McMurdie and Holmes, 2014).

## 3 Results

### 3.1 Sequencing depth and OTU assignment

A total of 907 OTUs (97% similarity) were found in the 48 samples used in this study, based on clustering of 358,465 ITS1 sequences (mean 7,468 quality-controlled reads per sample, see Supplementary Data Sheet 1 for full details). Taxonomic classifications of the OTUs based on the UNITE database are indicated in Supplementary Data Sheet 2.

### 3.2 Fungal community composition

**Figure 2:**
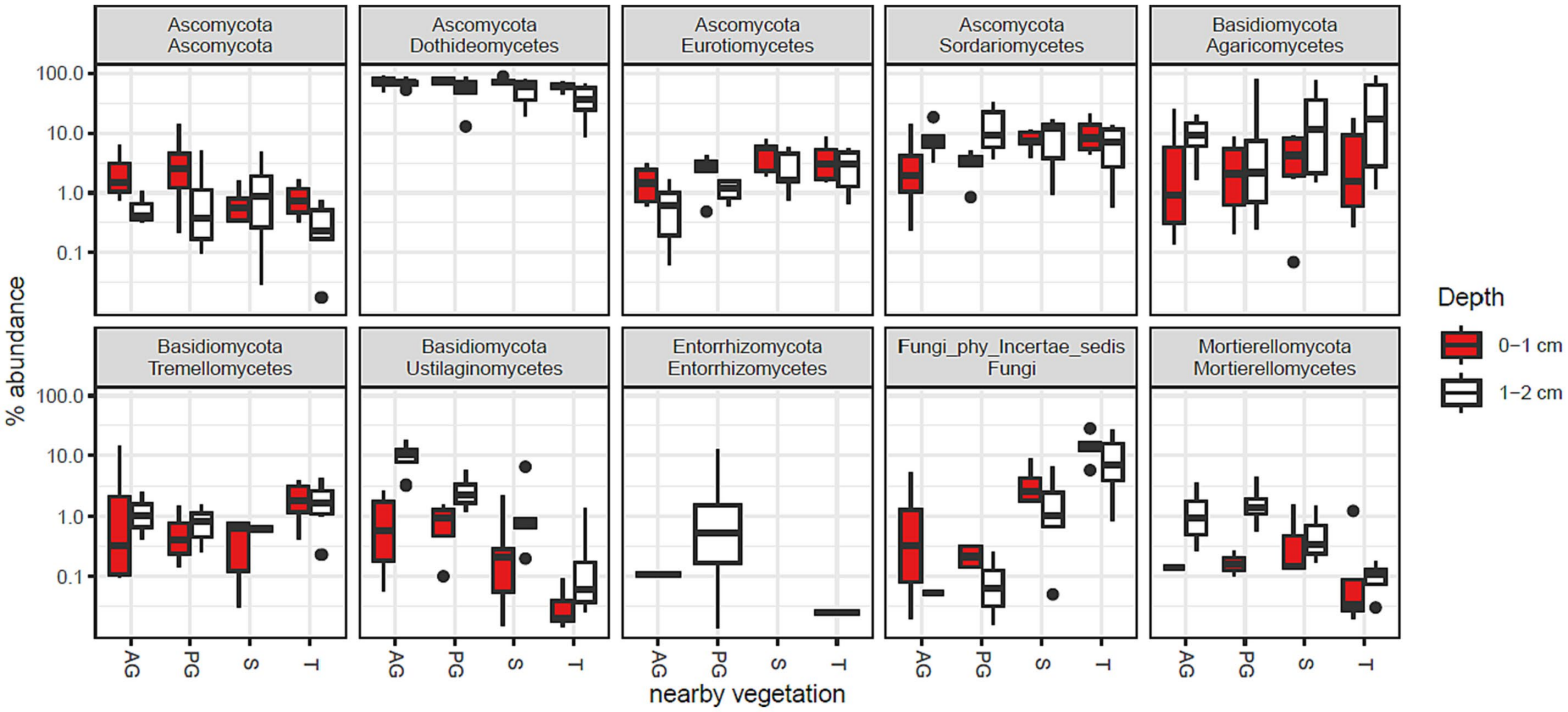
Fungal class abundance by vegetation zone and depth in Kalahari Sand biocrusts. Boxes represent the interquartile range (IQR), and error bars extend to the most extreme values within 1.5 * IQR of the box. Median values are shown as a line within the box and outliers are shown as black spots. Sample coding: AG, annual grass; PG, perennial grass; S, shrub; T, tree. The 10 most abundant classes are shown, accounting for 82% of sequence reads.

The phylum Ascomycota was abundant in all samples and was assigned to 75% of the fungal community in the whole study (Supplementary Data Sheet 2). Basidiomycota was the second most abundant phylum composing 17% of the community. The relative abundances of the 10 most abundant taxonomic classes are shown in Figure 2. In combination, these taxa account for 95% of the fungal community. The most abundant class was Dothideomycetes (62%), which was responsible for the overall dominance of the Ascomycota phylum.

### 3.3 Spatial structuring of fungal communities in relation to biocrust presence and depth

**Figure 3:**
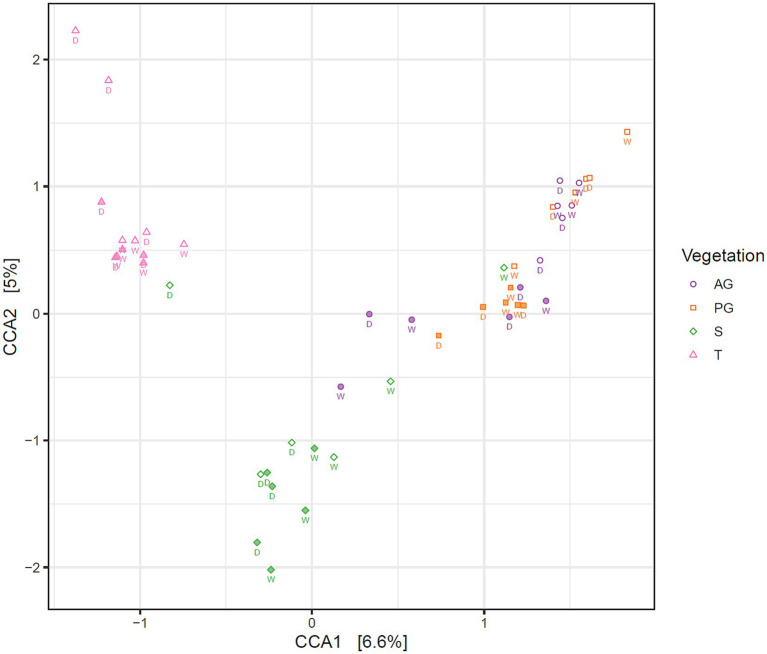
Correspondence analysis of the fungal community in Kalahari Sand biocrusts and immediately underneath biocrusts, constrained by vegetation and depth. Markers indicate the fungal communities of individual samples. Filled markers indicate soil surface communities (0–1 cm depth), open markers indicate subsoil communities (1–2 cm depth). Sample coding: AG, annual grass; PG, perennial grass; S, shrub; T, tree. The season of sample collection is indicated by W = wet season (March) and D = dry season (November).

A constrained correspondence analysis of the fungal community structure was performed as described for the bacterial analysis in Elliott et al. (2014). Results shown in Figure 3 revealed a three-way separation based on nearby vegetation (tree, shrub, or both grass species), and by soil depth restricted to the grass interspace areas only. Permutational analysis of variance indicated that fungal community structure differed significantly in relation to nearby vegetation and depth, but season did not significantly affect the community structure (Table 2).

**Table 2:** Permutational analysis of variance to identify significant differences in fungal community structure with respect to biocrust presence, depth, and season in Kalahari Sand. This table shows results for the community data at OTU level (97% similarity).

Factor	All samples^*^		Canopy samples+		Open samples+	
			(tree and shrub area)		(grass interspaces with cyanobacterial biocrust)	
	F	P	F	P	F	P
Vegetation	4.3	0.001	3.9	0.001	0.8	0.622
Depth	4.3	0.001	1.7	0.019	5.6	0.001
Season	0.8	0.785	1.0	0.468	0.8	0.728

Canopy areas lacked microbial phototrophs or had low levels of them and open areas had photosynthetic biocrusts rich in cyanobacteria. Results are based on Bray–Curtis dissimilarity matrices for species level OTUs (97% similarity). ^*^df = 3. + df = 1. F = pseudo-F value (effect size); P = probability.

### 3.4 Correlation between bacterial and fungal communities

Mantel tests were used to compare the bacterial (from Elliott et al., 2014) and fungal data (this study) community dissimilarity matrices to see if the community structure of fungi is correlated with the community structure of bacteria within biocrust samples and within non-biocrust samples. In surface soils from grass areas with biocrusts there was a significant correlation between bacterial and fungal communities (*p* = 0.001; r = 0.33). There was, however, no correlation between fungal and bacterial communities in the surface soil under trees and shrubs where no biocrust was present (*p* = 0.13; r = 0.14).

### 3.5 Abundance of fungi with respect to presence of cyanobacterial biocrust and depth

**Figure 4:**
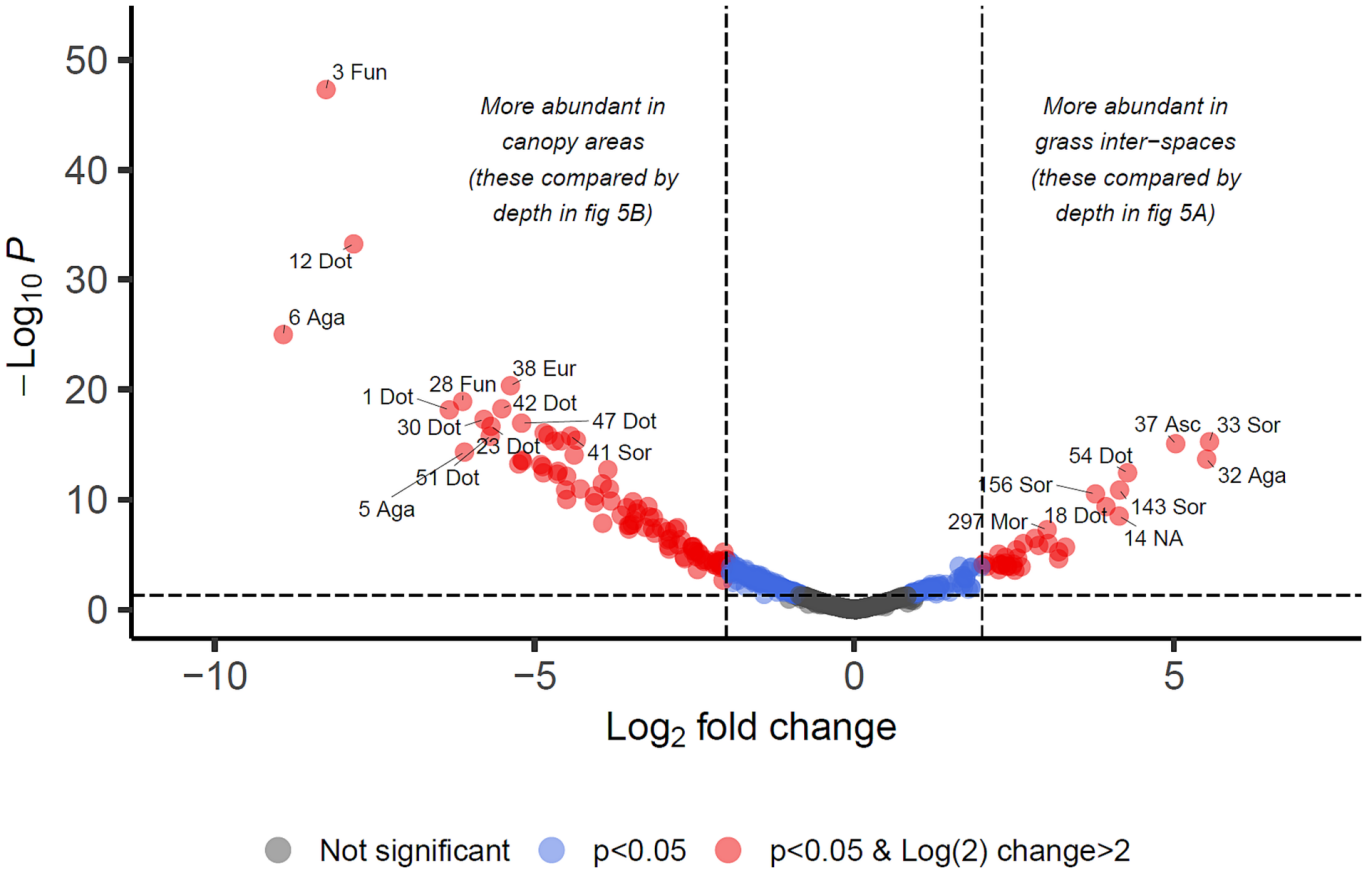
Differential abundance analysis of fungal OTUs in Kalahari Sand with relation to cover type of the land (biocrust of grass interspace or tree/shrub canopy). Guidelines on the plot indicate Log_2_ (=2) fold change in abundance and *p*-value < 0.05. Individual OTUs are shown as dots on the plot which are coloured according to those thresholds. Some of the OTUs are labelled with their OTU identification number and the first 3 letters of the taxonomic class to which they belong (Agaricomycetes, Dothideomycetes, Eurotiomycetes, Mortierellomycetes, Sordariomycetes, Ustilaginomycetes). Asc indicates uncertain taxonomic placement in the Ascomycota; Fun indicates uncertain taxonomic placement in the fungi. Full statistical results and taxonomy are provided in Supplementary Data Sheet 2.

**Figure 5:**
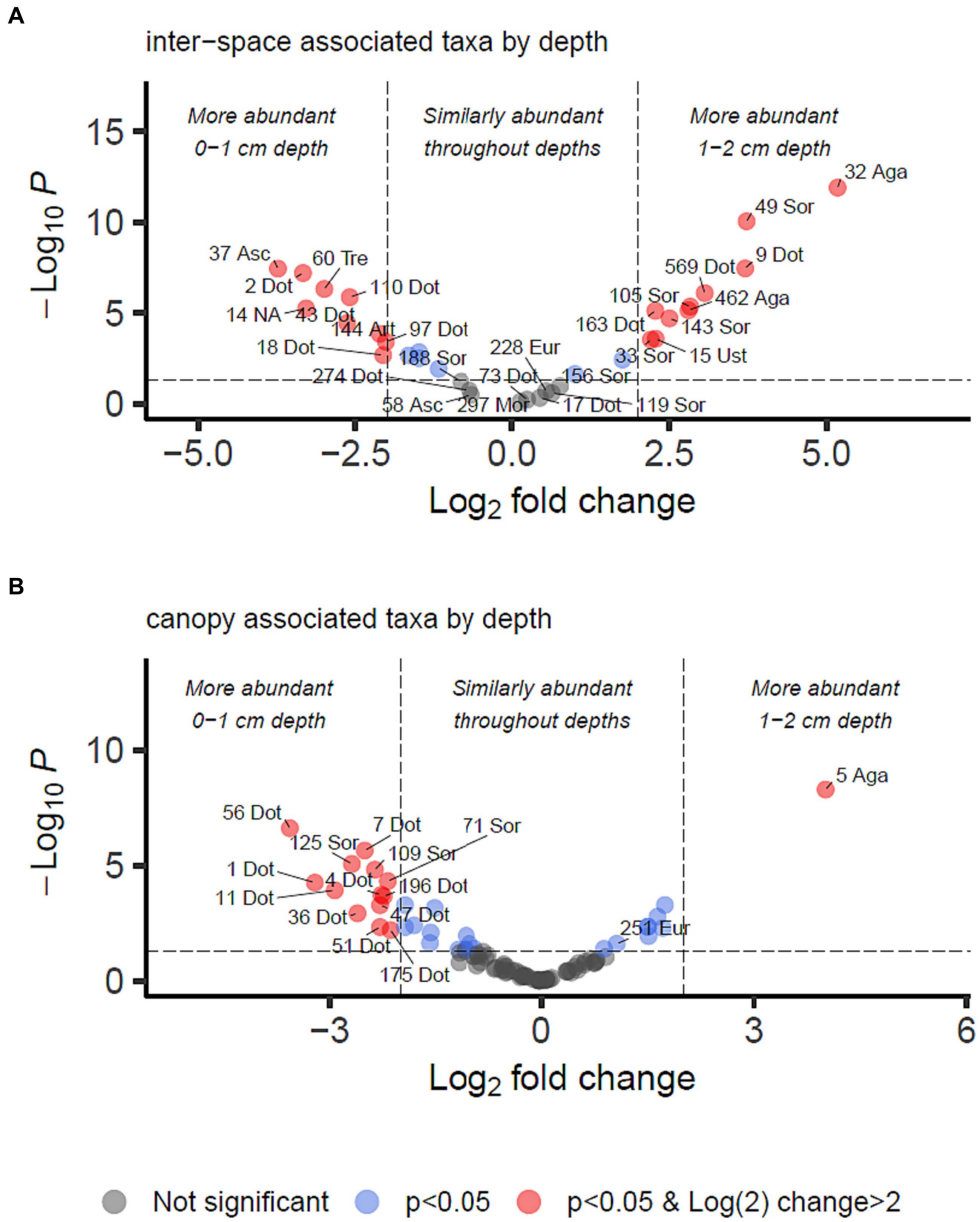
Differential abundance analysis of fungal OTUs associated with grass inter-spaces **(A)** or canopy cover **(B)** in Kalahari Sand (as identified in Figure 4), with relation to depth (0–1 or 1–2 cm). Guidelines on the plot indicate Log_2_ (=2) fold change in abundance and *p*-value < 0.05. Individual OTUs are shown as dots on the plot which are coloured according to those thresholds (see key). Some of the OTUs are labelled with their OTU identification number and the first 3 letters of the taxonomic class to which they belong (Agaricomycetes, Dothideomycetes, Eurotiomycetes, Mortierellomycetes, Sordariomycetes, Ustilaginomycetes). Asc indicates uncertain taxonomic placement in the Ascomycota; Fun indicates uncertain taxonomic placement in the fungi. Full statistical results and taxonomy are provided in Supplementary Data Sheet 2.

For identifying differential abundance of taxa using the DESeq2 method, we set a significance threshold of *p* < 0.05 and also a magnitude difference of 4-fold (log_2_ = 2) to minimise false positive results, as shown graphically in Figures 4, 5. Supporting data for these figures is supplied in Supplementary Data Sheet 2 from which can also be identified OTUs which may be of interest but did not meet the significance thresholds being reported in the manuscript text.

We identified 34 OTUs that were significantly associated with open areas (places where cyanobacterial biocrusts were present), and 91 OTUs significantly associated with canopy areas, from the total of 907 OTUs. We then took the subsets of OTUs identified for canopy and open areas, and checked for significant depth associations within those areas in order to test the hypotheses outlined in Table 1. From the grass-interspace OTUs we found 9 associated with the soil surface and 10 associated with the sub-surface soil (Figure 5). From canopy associated OTUs we found 13 associated with the soil surface and 1 associated with the sub-surface soil.

In Table 3, we present the taxonomy of OTUs which have distributions consistent with the hypothetical functions set out in Table 1. Most of the enriched taxa in biocrust surface soils belonged to the Ascomycota phylum, especially the orders Pleosporales and Mycosphaerellales in the class Dothideomycetes.

**Table 3:** Fungi found to be associated with grass interspaces (adjusted *p* < 0.05) that are either: **(A)** associated with the soil surface (biocrust); or **(B)** found similarly within and below the biocrust.

OTU	Taxonomy				Abundance %	
	Phylum	Class	Order	Family	0–1 cm	1–2 cm
**A. Taxonomy of OTUs associated with open areas and significantly more abundant in the surface layer (biocrust)**						
144	Ascomycota	Arthoniomycetes	Lichenostigmatales	Phaeococcomycetaceae	0.27	0.00
37	Ascomycota	unidentified	unidentified	unidentified	2.41	0.25
97	Ascomycota	Dothideomycetes	Capnodiales	unidentified	0.63	0.04
110	Ascomycota	Dothideomycetes	Mycosphaerellales	Extremaceae	0.43	0.08
18	Ascomycota	Dothideomycetes	Mycosphaerellales	Teratosphaeriaceae	6.46	3.03
2	Ascomycota	Dothideomycetes	Pleosporales	Didymellaceae	21.64	3.66
43	Ascomycota	Dothideomycetes	Pleosporales	Periconiaceae	0.93	0.25
60	Basidiomycota	Tremellomycetes	Tremellales	Rhynchogastremataceae	1.49	0.03
14	unidentified	unidentified	unidentified	unidentified	6.21	1.15
**B. Taxonomy of OTUs associated with open areas which are similarly abundant in the surface layer (biocrust) and beneath it**						
58	Ascomycota	unidentified	unidentified	unidentified	0.37	0.18
274	Ascomycota	Dothideomycetes	Mycosphaerellales	Teratosphaeriaceae	0.11	0.03
73	Ascomycota	Dothideomycetes	Pleosporales	Periconiaceae	0.27	0.38
17	Ascomycota	Dothideomycetes	Pleosporales	unidentified	1.98	3.22
228	Ascomycota	Eurotiomycetes	Eurotiales	Aspergillaceae	0.02	0.12
188	Ascomycota	Sordariomycetes	Coniochaetales	Coniochaetaceae	0.15	0.06
119	Ascomycota	Sordariomycetes	Sordariales	Chaetomiaceae	0.05	0.59
156	Ascomycota	Sordariomycetes	Sordariales	Chaetomiaceae	0.13	0.36
297	Mortierellomycota	Mortierellomycetes	Mortierellales	Mortierellaceae	0.04	0.27

These taxa exhibit spatial distributions consistent with short-and long-range interactions with biocrusts as hypothesised in Table 1.

To identify biocrust associated fungi which may hypothetically be delivering resource transportation functions extending beyond the soil surface, we took the intersection of OTUs significantly more abundant in grass interspaces but with no significant difference in abundance between crust and subsoil in the grass interspace zone. These OTUs are therefore associated with areas having a biocrust, but they are not confined to the biocrust surface layer. There were 9 OTUs matching these criteria (Table 3), of which three belonged to the class Dothideomycetes which was also the most abundant class.

## 4 Discussion

We characterised the fungal communities within and beneath biocrusts of Kalahari Sand soils in Botswana, addressing the lack of biocrust fungal community data globally, and providing the first report of fungal community composition in biocrusts of Africa using high-throughput DNA sequencing approaches. By using a fine spatial resolution of sampling, we show that the soil surface (0–1 cm) fungal communities of biocrusts are distinct from the immediate sub-surface communities (1–2 cm). Furthermore, we demonstrate that within the biocrust (0–1 cm), bacterial and fungal communities do not vary independently of each other, but they are correlated (i.e., a change in the bacterial community is reflected by a corresponding non-random change in the fungal community). This correlation of bacterial and fungal communities occurs only in the biocrusts and not in the soil beneath biocrusts or areas lacking biocrusts, thus indicating the existence of cross-kingdom biological interactions within biocrusts.

At the OTU level we found that biocrust fungal communities in grass interspaces differ from communities in the immediate soil beneath the biocrust (Table 2), showing that biocrusts contain specifically adapted fungal communities. In soil surfaces under trees and shrubs there was little or no capacity for *in-situ* photosynthesis because cyanobacteria and algae are absent or rare (Thomas and Dougill, 2007; Elliott et al., 2014). In these areas, vertical stratification of the fungal community was less pronounced, and fungal community structure was more likely driven by the composition of nearby vegetation (Table 2). This result may reflect soil microbial community adaptations to different co-occurring routes of carbon fixation into biomass and has potential implications for the fungal loop model. If photosynthesis within biocrusts is sufficient then there is no selective pressure to establish a mutualism for obtaining carbon from plants, and furthermore doing so may be deleterious to the biocrust if it benefits the plant to the extent of causing shading of the cyanobacteria. The results presented in this research support such a scenario, adding to the evidence of Dettweiler-Robinson et al. (2020) which found little response of biocrusts when fungal connections to plants were interrupted.

Prior studies comparing the fungal composition of biocrusts and soil beneath biocrusts include Steven et al. (2014), Pombubpa et al. (2020), and Zhang et al. (2022). They have shown that biocrusts have a distinct fungal composition compared to the sub-surface soil under a variety of scenarios which indicates a general rule that probably applies to all biocrusts. In this work we extend those findings by using a fine scale of sampling and performing a novel analysis of fungal distribution which enabled us to identify taxa that may be associated with hypothesised functions. From a total of 907 OTUs, we identified 34 that were significantly more abundant in open areas harbouring biocrusts, of which 9 were more abundant in the biocrust itself (Table 3). The taxa identified in Table 3 are potentially biocrust associated based on their distribution being consistent with hypothetical short-range and long-range interactions. Most of the candidate biocrust specific taxa are ascomycetes in the class Dothidiomycetes. Similar fungi have previously been found to be abundant in biocrusts (e.g., Green et al., 2008; Abed et al., 2013; Steven et al., 2014), and they have adaptations for survival in extreme conditions (Ruibal et al., 2009). It should be noted that no primer set can faithfully capture the real biological diversity of complex soil samples, and the results reported here are interpreted with the knowledge of potential biases. The primers used in this study are known to favour the detection of Ascomycota and Basidiomycota, which may contribute to the apparent high abundance of these groups in the results.

Some Pleosporales are described as dark septate endophytic fungi (DSE), because of their melanised septa and tendency to grow endophytically with plants. Melanin production is of interest in relation to biocrusts because of its probable function in protecting biocrusts from UV damage (Bates and Garcia-Pichel, 2009). DSE are a very abundant group, however their roles are mostly unknown (Mandyam and Jumpponen, 2005; Knapp et al., 2015), which raises the possibility of biocrust related roles which have been suggested by Green et al. (2008). We did not specifically identify which of the Pleosporales sequences obtained in this study belong to the DSE fungi, but due to the abundance of this group in similar ecosystems we conclude that it is likely that DSE are a subset of the Pleosporales sequences observed.

Circumstantial support for a role for Pleosporales in biocrust ecology is provided by numerous reports of DSE fungi being abundant in dryland areas (Knapp et al., 2015), especially grasslands (e.g., Porras-Alfaro et al., 2011), but also on rock surfaces (Ruibal et al., 2009), all of which are locations where biocrusts are abundant. Our data add to this by showing that certain Pleosporales OTUs are significantly associated with biocrusts and not simply existing in the vicinity of biocrusts, and that they participate in fungal communities which are correlated with the phototrophic communities of biocrusts (e.g., Tables 2, 3).

The fungal loop model of Collins et al. (2008) proposes a role of fungi in nutrient transportation in drylands. Green et al. (2008) and studies referenced therein demonstrate carbon and nitrogen translocation between biocrusts (0–1 cm depth) and plants, with fungi in the order Pleosporales thought to be dominant in this process. Whilst some of the Pleosporales taxa identified in this study were specifically associated with the biocrust layer, we have also identified other taxa including Pleosporales species which are associated with the whole depth profile in biocrust areas (0–2 cm depth). These fungal taxa (Table 3) are connected to biocrusts whilst simultaneously occupying compartments of the soil profile with different resource profiles, hence they are likely candidates for performing the translocation functions predicted by the fungal loop model. Whilst we do not track individual hyphae or verify the connection to plant roots, these results nevertheless provide progress towards testing the criteria of the fungal loop model proposed by Rudgers et al. (2018).

The relationship between soil fungal community and above-ground plant composition is well known (e.g., van der Heijden et al., 2008; Prober et al., 2015), and we hypothesised that biocrusts similarly have characteristic fungal communities associated with them. This is supported by our finding that bacterial and fungal community distance matrices correlate with each other in the open grass interspaces where cyanobacterial biocrusts are present. In contrast, no such correlation of bacterial and fungal communities was found in the tree and shrub zones that lack cyanobacterial biocrusts, even though these areas have previously been shown to have distinctive biocrust-like heterotrophic bacterial communities at the soil surface (Elliott et al., 2014). These results show that bacterial and fungal communities of cyanobacterial biocrusts are ecologically connected, implying that cross-kingdom biotic filtering may be a driving factor of community assembly in biocrusts and hence of functional significance. We propose that different fungi probably engage in short-range and long-range interactions with dryland soil surface bacteria (e.g. Table 1) and this may have important implications for our understanding of dryland soil function and ecology, including the fungal loop model. We have identified some taxa which merit further investigation owing to their differential abundance with respect to depth or presence of cyanobacteria. To facilitate deeper analysis of individual interactions in our data, we have supplied Supplementary material showing the detailed differential abundance analysis results, plus code supporting the analyses (see section 12). Most notably we highlight the striking abundance and distribution of Ascomycete fungi in the class Dothidiomycetes and conclude that this diverse group is likely to contain many taxa specifically adapted for various niches in biocrusts and play an important role in dryland ecosystem productivity.

## Data availability statement

Sequence data and metadata are available on the MG-RAST metagenomics analysis server (Meyer et al., 2008) in project 6691, and on the NCBI sequence read archive (SRA) in BioProject PRJNA305652 at https://www.ncbi.nlm.nih.gov/bioproject/PRJNA305652. Code used for the analysis presented in this paper is available at https://github.com/davidelliott/kalahari-fungi.

## Author contributions

AT, RS, SH, and DE planned and designed the research. DE performed the experiments and analysed the data. AT and DE conducted the fieldwork and wrote the manuscript. RS and SH edited the manuscript and contributed ideas. All authors contributed to the article and approved the submitted version.

## In memoriam

We would like to thank the late Jill Thomas of Berrybush farm for her considerable support and access to her land. We will be forever grateful for her kindness and for passing on some of her considerable knowledge of the Kalahari environment.
